# Supplementary material for: HiPhase: jointly phasing small, structural, and tandem repeat variants from HiFi sequencing
Source: Bioinformatics. 2024 Jan 25;40(2):btae042. doi: 10.1093/bioinformatics/btae042 (PMC10868326; doi:10.1093/bioinformatics/btae042)
Supplement: btae042_Supplementary_Data [file btae042_supplementary_data.pdf]

Supplemental Material for "HiPhase: Jointly phasing small and structural variants from HiFi sequencing"

James M. Holt, Christopher T. Saunders, William J. Rowell, Zev Kronenberg, Aaron M. Wenger, and Michael Eberle

PacBio, Menlo Park, CA USA

18th Jan, 2024

Contents

1 Data details **3**

1.1 Benchmark files . . . 3
1.2 Sequencing data . . . 3
1.3 Tool and pipeline versions . . . 4
1.4 Tool definitions . . . 4
1.4.1 WhatsHap . . . 4
1.4.2 WhatsHap (optimized) . . . 6
1.4.3 HiPhase (no SV) . . . 6
1.4.4 HiPhase . . . 6

2 Additional Results **7**

2.1 All methods comparison . . . 7
2.2 Overall summary figure . . . 7
2.3 Sequel II system summary metrics . . . 7
2.4 Assembly-based comparisons . . . 10
2.5 Coverage experiment . . . 12
2.5.1 HiPhase coverage performance . . . 13
2.5.2 WhatsHap coverage performance . . . 13
2.6 Supplemental mapping experiment . . . 13
2.7 Phased variant differences . . . 13

3 Result metrics **13**

3.1 Metric definitions . . . 16
3.1.1 Error metrics . . . 16
3.1.2 Phase block metrics . . . 16
3.2 Error metric command templates . . . 16
3.3 Phase block metric command templates . . . 17
3.3.1 NG50 and number of phased variants . . . 17
3.3.2 NGC50 and "Fully phased genes" . . . 18
3.3.3 Number of phased structural and tandem repeat variants . . . 19
3.4 Computational resource metrics . . . 19

4 Deep Methods **20**
4.1 Phase block generation . . . 20
4.2 Allele assignment . . . 21
4.2.1 Local re-alignment . . . 22
4.2.2 Global re-alignment . . . 22
4.2.3 Collapsing mappings . . . 23
4.3 Diplotype Solving . . . 23
4.3.1 A* phasing algorithm . . . 23
4.3.2 Defining the search space . . . 24
4.3.3 Defining the cost structure . . . 25
4.3.4 Defining the heuristic . . . 25
4.4 Algorithm Statistics . . . 26

1 Data details

1.1 Benchmark files

We benchmarked against the Genome in a Bottle (GIAB) consortium phased variant calls v4.2.1 (Wagner et al., 2022). Table 1 contains direct links to the files downloaded from GIAB for each of the three benchmark samples.

Table 1: Links to the phased benchmark set, GIAB v4.2.1, used for each sample.

Sample	Benchmark file
HG001	https://ftp-trace.ncbi.nlm.nih.gov/ReferenceSamples/giab/release/NA12878_HG001/NISTv4.2.1/GRCh38/SupplementaryFiles/HG001_GRCh38_1_22_v4.2.1_benchmark_hifiasm_v11_phasetransfer.vcf.gz
HG002	https://ftp-trace.ncbi.nlm.nih.gov/ReferenceSamples/giab/release/AshkenazimTrio/HG002_NA24385_son/NISTv4.2.1/GRCh38/SupplementaryFiles/HG002_GRCh38_1_22_v4.2.1_benchmark_hifiasm_v11_phasetransfer.vcf.gz
HG005	https://ftp-trace.ncbi.nlm.nih.gov/ReferenceSamples/giab/release/ChineseTrio/HG005_NA24631_son/NISTv4.2.1/GRCh38/SupplementaryFiles/HG005_GRCh38_1_22_v4.2.1_highconf_hifiasm_v11_phasetransfer.vcf.gz

1.2 Sequencing data

Links to the sequencing data used for all analyses can be found in Table 2. Datasets from the Revio system are used in the main manuscript. Additional datasets from the Sequel II system from the Genome in a Bottle consortium (Zook et al., 2016) are only analyzed within this supplement. For HG002 and HG005 datasets from Sequel II systems, we used a subset of all available sequencing data to achieve approximately 30x read depth after alignment. All other datasets used every SMRT Cell that was available. Both unphased and phased VCF files for WhatsHap and HiPhase are available for the three Revio HG002 replicates at the URL specified in Table 4.

Table 2: Table with links to sequencing datasets used in this document. Datasets marked with an asterisk (*) used a subset of all available movies to reach approximately 30x. These datasets have the exact SMRT Cells used listed. All others are marked with "All" and the number of SMRT Cells available for use.

System	Dataset	SMRT Cells used	URL
Sequel II	HG001	All (6)	https://ftp-trace.ncbi.nlm.nih.gov/ReferenceSamples/giab/data/NA12878/HudsonAlpha_PacBio_CCS/
	HG002*	m64012_190920_173625<br>m64012_190921_234837<br>m64015_190920_185703	https://ftp-trace.ncbi.nlm.nih.gov/ReferenceSamples/giab/data/AshkenazimTrio/HG002_NA24385_son/PacBio_CCS_15kb_20kb_chemistry2/reads/
	HG005*	m64017_200723_190224<br>m64109_200304_195708<br>m64109_200309_192110<br>m64109_200311_013444	https://ftp-trace.ncbi.nlm.nih.gov/ReferenceSamples/giab/data/ChineseTrio/HG005_NA24631_son/HudsonAlpha_PacBio_CCS/
Revio	HG002-rep1	All (1)	https://downloads.pacbcloud.com/public/revio/2022Q4/HG002-rep1/
	HG002-rep2	All (1)	https://downloads.pacbcloud.com/public/revio/2022Q4/HG002-rep2/
	HG002-rep3	All (1)	https://downloads.pacbcloud.com/public/revio/2022Q4/HG002-rep3/

Table 3: Summary metrics for each dataset used in our analysis. Mean coverage was gathered after alignment from `mosdepth` (Pedersen and Quinlan, 2017a). Read length statistics were collected from `fastleng`.

System	Dataset	Mean coverage	Mean read length	N25 read length	N10 read length
Sequel II	HG001	25.99x	17,539	19,800	23,502
	HG002	28.40x	12,858	13,702	14,516
	HG005	31.81x	17,390	19,556	21,347
Revio	HG002-rep1	32.38x	15,474	20,696	24,342
	HG002-rep2	29.59x	15,296	20,465	24,027
	HG002-rep3	27.96x	15,247	20,407	24,072

Summary statistics for each analyzed dataset are available in Table 3. Each dataset has approximately 30x sequencing depth. The three HG002 replicates sequenced on the Revio system have lower mean read lengths than HG001 or HG005, but they have a tail of longer reads as evidenced by higher N10 and N25 read lengths. Compared to the other datasets, the HG002 dataset sequenced on the Sequel II system has noticeable shorter read lengths.

1.3 Tool and pipeline versions

Table 4 contains the versions and links for all tools and pipelines that were used to generate results in this document. Additionally, a link to the unphased and phased outputs for the main document are available in the "VCF data bundle" link.

1.4 Tool definitions

The following list describes each of the tools used in our analysis, both in the main document and this supplement.

- WhatsHap - The current recommended approach for WhatsHap (Patterson et al., 2015) on HiFi datasets. It phases small variants from DeepVariant, including both SNVs and indels. This is the WhatsHap approach used for analyses in the primary document.
- WhatsHap (optimized) - Through experimentation, we found that allowing WhatsHap to distrust the genotypes tended to reduce the number of errors generated in the result at the cost of phase block length. However, this has the additional side-effect of allowing the tool to change heterozygous genotypes to homozygous in the output VCF files.
- HiPhase (no SV) - The method described in this paper when provided only the DeepVariant calls (i.e., no structural variants or short tandem repeat variants). This approach uses local re-alignment for allele assignment and is the recommend approach for phasing only small variants.
- HiPhase - This approach jointly phases DeepVariant calls with the large insertion and deletion calls from `pbsv` as well as short tandem repeat calls from TRGT. This method uses global re-alignment and is our recommended approach for phasing with structural variants. This is the HiPhase approach used for analyses in the primary document.

The following sections contain the command templates used for each tool in our comparative analysis.

1.4.1 WhatsHap

Program 1 contains the template we used for WhatsHap. For both WhatsHap variants, we parallelized by running one chromosome per job. This is specified in the {`wildcards.chrom`} parameter. The only other notable parameter is `--indel`, which enables the phasing of indels with SNVs.

Table 4: Tool versions and URLs. This table contains primary tools used in this document as well as pipeline resources more upstream processing. Each tool or pipeline is tagged with the version used in this document and a link to the public repository.

**Tool / method name**	**Version(s)**	**URL**
HiPhase (this paper)	v1.0.0	https://github.com/PacificBiosciences/HiPhase
WhatsHap (Patterson et al., 2015)	v1.4	https://github.com/whatshap/whatshap
Secondary Pipeline	–	https://github.com/PacificBiosciences/pb-human-wgs-workflow-snakemake
Reference genome	GRCh38 no alt analysis set	ftp://ftp.ncbi.nlm.nih.gov/genomes/all/GCA/000/001/405/GCA_000001405.15_GRCh38/seqs_for_alignment_pipelines.ucsc_ids/GCA_000001405.15_GRCh38_no_alt_analysis_set.fna.gz
pbmm2	v1.4.0	https://github.com/PacificBiosciences/pbmm2
DeepVariant (Poplin et al., 2018)	v1.3.0 (Sequel II system)<br>v.1.5.0 (Revio system)	https://github.com/google/deepvariant
pbsv	v2.8.0	https://github.com/PacificBiosciences/pbsv
TRGT (Dolzhenko et al., 2023)	v0.5.0	https://github.com/PacificBiosciences/trgt
mosdepth (Pedersen and Quinlan, 2017a)	v0.2.9	https://github.com/brentp/mosdepth
fastleng	v0.2.0	https://github.com/HudsonAlpha/rust-fastleng
vcfdist (Dunn and Narayanasamy, 2023)	v2.3.1	https://github.com/TimD1/vcfdist
VCF data bundle	v1.0.0	https://doi.org/10.5281/zenodo.10233350

Program 1: Command line template for the baseline WhatsHap phasing method.

```
whatshap phase \
  --indel \
  --ignore-read-groups \
  --chromosome {wildcards.chrom} \
  --output {output.vcf} \
  --reference {input.reference} \
  {input.vcf} \
  {input.bams}
```

Program 2: Command line template for the WhatsHap (optimized) phasing method.

```
whatshap phase \
  --indel \
  --distrust-genotypes \
  --ignore-read-groups \
  --chromosome {wildcards.chrom} \
  --output {output.vcf} \
  --reference {input.reference} \
  {input.vcf} \
  {input.bams}
```

Program 3: Command line template for the HiPhase (no SV) phasing method.

```
hiphase \
  --threads 16 \
  --reference {input.reference} \
  --bam {input.bam1} \
  ...
  --bam {input.bamN} \
  --vcf {input.vcf} \
  --output-vcf {output.vcf} \
  --stats-file {output.stats} \
  --blocks-file {output.blocks} \
  --summary-file {output.summary}
```

1.4.2 WhatsHap (optimized)

Program 2 is identical to the above, but with the added `---distrust-genotypes` option. This option will modify the algorithm to allow it to convert heterozygous calls into homozygous calls. We found that this typically reduces the number of errors generated by WhatsHap and reduces phase block length (NG50), but this leads to an overall increase in corrected phase block length (NGC50). Additionally, it has the side effect of modifying the output variants to homozygous (0/0 or 1/1) instead of leaving them as unphased heterozygous calls.

1.4.3 HiPhase (no SV)

The command in Program 3 is the baseline HiPhase method that is most comparable to WhatsHap because it only phases small variants. It is also the recommended approach if only a small variant call file is available. Each BAM is specified via the `--bam` parameter (multiple times if the data is stored in multiple BAM files). Multi-threading is enabled via `--threads` and multiple statistics files are also output while running (`--stats-file`, `--blocks-file`, and `--summary-file`). We note that similar to "WhatsHap (optimized)", HiPhase is allowed to convert heterozygous calls to homozygous if that leads to a more optimal solution. However, in contrast to WhatsHap, these variants are not converted in the output and are instead left as unphased heterozygous calls in the VCF.

1.4.4 HiPhase

Program 4 is the *recommended* way to use HiPhase for jointly phasing small variants with structural variants and short tandem repeats. The main changes relative to the above command are 1) the addition of a two additional input and output structural variant and tandem repeat VCFs and 2) the enabling of global re-alignment with the `--global-realignment-cputime` parameter. Note that variants fully contained within

Program 4: Command line template for the HiPhase phasing method.

```
hiphase \
  --threads 16 \
  --global-realignment-cputime 300 \
  --reference {input.reference} \
  --bam {input.bam1} \
  ...
  --bam {input.bamN} \
  --vcf {input.vcf} \
  --output-vcf {output.vcf} \
  --vcf {input.pbsv_vcf} \
  --output-vcf {output.pbsv_vcf} \
  --vcf {input.trgt_vcf} \
  --output-vcf {output.trgt_vcf} \
  --stats-file {output.stats} \
  --blocks-file {output.blocks} \
  --summary-file {output.summary}
```

an STR variants are effectively masked from the inputs and left unphased. This is because a single STR call from TRGT frequently encapsulates multiple calls from a small variant VCF file.

2 Additional Results

In the main document, we focus on the latest sequencing data generated by the Revio system. However, the publicly available samples that also have a GIAB benchmark are limited to HG002. In these sections, we provide extended results for the Revio system datasets, and we also provide results for Sequel II system datasets that extend to HG001, HG002, and HG005 samples.

2.1 All methods comparison

Figure 1 and Table 5 show the summary comparison for all tool definitions in this supplement (see Section 1.4) restricted to datasets from the Revio system. Compared to the current practice ("WhatsHap"), both HiPhase conditions generated fewer errors (switches and flips) and longer corrected phase blocks (NGC50). Additionally, there is a small but noticeable reduction in errors when SVs and STRs are provided to HiPhase. We also note that these results demonstrate the potential benefit of the "WhatsHap (optimized)" mode relative to the current WhatsHap. Despite decreasing NG50, the positive effects of reducing errors leads to an overall improvement in NGC50.

2.2 Overall summary figure

Figure 2 shows the summary comparison for all datasets split by Sequel II or Revio systems and tool definitions. All methods on Revio system datasets produced higher NGC50 than the Sequel II system comparators. Additionally, HiPhase runs had a comparable switch and flip counts, whereas WhatsHap tended to increase in error on Revio system datasets.

2.3 Sequel II system summary metrics

Table 6 shows the more detailed summary results for Sequel II system datasets when evaluated on the four tool definitions. The patterns shown in these results generally reflect the same patterns observed in Revio system datasets.

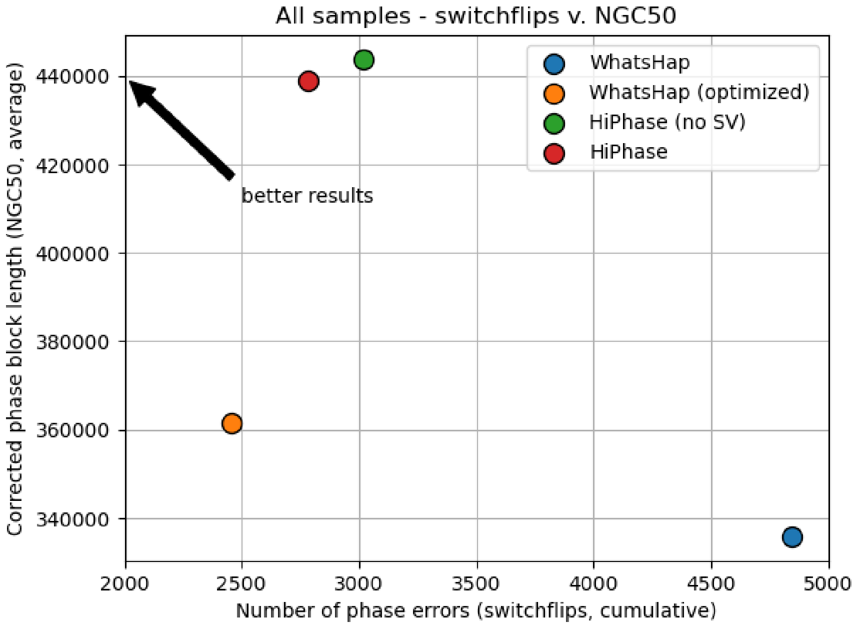

Figure 1: Summary figure showing switchflips v. NGC50 for all methods. Switchflips represent raw error counts whereas NG50 represents the length of error-free portions of the constructed phase blocks. HiPhase generated fewer switchflip errors than the primary comparator "WhatsHap", but more errors than the "WhatsHap (optimized)" version. However, NGC50 values were consistently higher for HiPhase than either WhatsHap method.

Table 5: Summary metrics for Revio system datasets (three replicates of HG002) for each method. Switches, flips, hamming distance, and NGC50 are measures of error. NG50 and NGC50 are measures of phase block length. Number of phased variants is broken into "all" category, the structural variants (SVs) from `pbsv`, and the short tandem repeat variants (STRs) from TRGT. Switchflips, hamming distance, and the number of phased variants are totaled across all datasets (sum), whereas NG50, NGC50, and genes full phased are averaged (mean). Metrics marked with an asterisk (*) are derived from tool-specific sources, details of which are available in Section 3.1. **Bolded** values represent the best performance in the row. Similar to the results from the main document, HiPhase outperforms all other methods with the exception of switches and hamming distance from "WhatsHap (optimized)".

**Metric**	**WhatsHap**	**WhatsHap (optimized)**	**HiPhase (no SV)**	**HiPhase**
Switches	3,039	**2,115**	2,742	2,523
Flips	1,805	342	274	**263**
Hamming distance	148,201	**91,356**	166,184	138,854
NG50*	406,912	393,252	**491,782**	479,953
NGC50	335,787	361,338	**443,804**	438,834
Fully phased genes	87.0%	85.9%	**94.0%**	93.8%
Number of phased variants (all)*	8,807,866	8,652,027	9,128,275	**9,159,840**
Number of phased SVs (> 50 bp)	N/A	N/A	N/A	**33,482**
Number of phased STRs	N/A	N/A	N/A	**204,783**

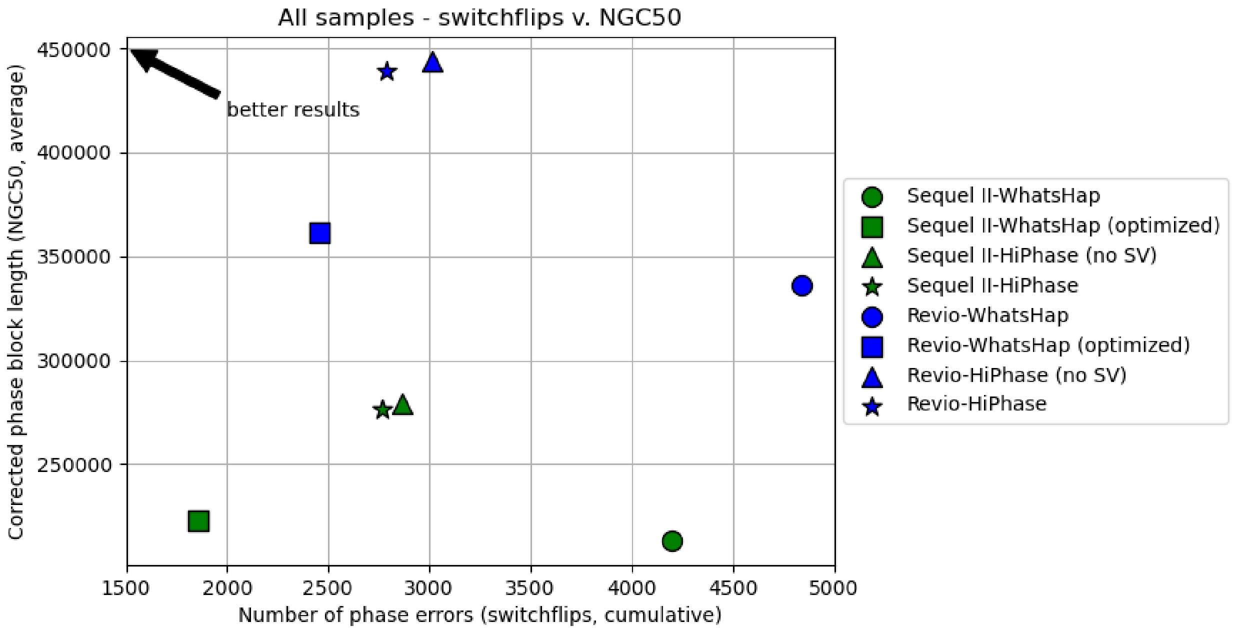

Figure 2: Summary figure showing switchflips v. NGC50 for all conditions ({system}-{tool}). Ideally, there are higher NGC50 values and lower switchflip errors. HiPhase generated fewer switchflip errors than the primary comparator "WhatsHap", but more errors than the "WhatsHap (optimized)" version. However, NGC50 values were consistently higher for HiPhase than either WhatsHap method. Additionally, HiPhase runs on Revio system datasets generated longer NGC50s with comparable error profiles to those from Sequel II systems. In constrast, WhatsHap tended to generate more errors on Revio system datasets than the Sequel II system datasets.

Table 6: Summary metrics for Sequel II system datasets (HG001, HG002, and HG005) for each method. Switches, flips, hamming distance, and NGC50 are measures of error. NG50 and NGC50 are measures of phase block length. Number of phased variants is broken into "all" category, the structural variants (SVs) from `pbsv`, and the short tandem repeat variants (STRs) from TRGT. Switchflips, hamming distance, and the number of phased variants are totaled across all datasets (sum), whereas NG50, NGC50, and genes full phased are averaged (mean). Metrics marked with an asterisk (*) are derived from tool-specific sources, details of which are available in the Section 3.1. **Bolded** values represent the best performance in the row.

**Metric**	**WhatsHap**	**WhatsHap (optimized)**	**HiPhase (no SV)**	**HiPhase**
Switches	2,624	**1,663**	2,748	2,670
Flips	1,572	193	118	**94**
Hamming distance	110,379	**71,497**	122,609	122,007
NG50*	248,730	238,626	**303,282**	302,062
NGC50	213,308	223,106	**278,805**	276,227
Fully phased genes	84.6%	83.0%	**91.7%**	91.4%
Number of phased variants (all)*	8,608,868	8,467,279	8,852,826	**8,871,253**
Number of phased SVs (> 50 bp)	N/A	N/A	N/A	**33,321**
Number of phased STRs	N/A	N/A	N/A	**203,389**

2.4 Assembly-based comparisons

While the GIAB truth sets are the primary established datasets for benchmarking phasing, they do not include larger structural variants in the benchmark. Recently, a group is working to establish an assembly-based phasing benchmark for HG002 that includes larger structural variants in the dataset. While this data is not formally published, the v0.9 preliminary benchmark has been made available at this link: https://ftp-trace.ncbi.nlm.nih.gov/ReferenceSamples/giab/data/AshkenazimTrio/analysis/NIST_HG002_DraftBenchmark_defrabbV0.011-20230725/.

With the inclusion of these larger events, we needed a new comparator tool as `whatshap compare` was not designed with these events in mind. Fortunately, a new tool called `vcfdist` is designed to jointly evaluate smaller variants (SNV/indel) and larger structural variants. However, this tool is less established in the community and still under active development for both accuracy and run-time. While both the new benchmark and evaluation tool are unpublished and under development, the preliminary results below help demonstrate the performance of HiPhase with structural and tandem repeat variants.

We ran comparisons of the WhatsHap, HiPhase (no SV), and HiPhase outputs using the following conditions which are summarized in Table 7:

1. WHC (WhatsHap Compare) - The established GIAB v4.2.1 HG002 is used as the benchmark set, only small variants are used in the evaluation, and `whatshap compare` is used to assess switches and flips. This is the baseline approach that has been used throughout the main manuscript and this document.

2. ASM - The new assembly-based v0.9 HG002 is used as the benchmark set, only small variants are used in the evaluation, and `whatshap compare` is used to assess switches and flips. We do not recommend this approach, but show it to demonstrate the effect of modifying just the benchmark set to the new assembly-based benchmark.

3. ASM-all - The new assembly-based v0.9 HG002 is used as the benchmark set, all variant VCFs are merged prior to evaluation (see Program 5), and `whatshap compare` is used to assess switches and flips. Only HiPhase is evaluated as it is the only one incorporating structural and tandem repeat variations. Note that while this incorporate larger events into the evaluation, `whatshap compare` was not designed with this larger variation in mind, making it difficult to compare the phase accuracy for larger events.

4. vcfdist - The new assembly-based v0.9 HG002 is used as the benchmark set, only small variants are used in the evaluation, and `vcfdist` v2.3.1 is used to assess switches and flips (see Program 6).

5. vcfdist-all - The new assembly-based v0.9 HG002 is used as the benchmark set, all variant VCFs are merged prior to evaluation (see Program 5), and `vcfdist` v2.3.1 is used to assess switches and flips (see Program 6). Only HiPhase is evaluated as it is the only one incorporating structural and tandem repeat variations. In constrast to `whatshap compare`, this approach is designed to handle larger events, such as SVs and STRs. When `vcfdist` becomes more established and computationally efficient, we expect this (or a similar approach) to be the recommended method for evaluating joint phasing across small, structural, and tandem repeat variants.

Table 8 shows the results of this experiment. Due to the preliminary nature of both the benchmark and the evaluation tool, we encourage readers not to view these results as precise metrics, but rather as relative trends. We highlight some of these trends below:

1. Switches are relatively consistent when changing benchmark sets - Changing just the benchmark set (comparing WHC to ASM), we see that switches are relatively consistent. In contrast, flips seems to increase significantly, especially for WhatsHap. We suspect this is an artifact of the evaluation tool with a benchmark including SVs as opposed to a true measure.

2. Vcfdist reports fewer switches and flips on the ASM benchmark - When we also change the evaluation tool, the number of switches drops across all conditions (comparing vcfdist to ASM). Additionally, flips for HiPhase conditions are relatively stable while the WhatsHap condition seems to have the excessive flip count corrected.

Table 7: Summary of evaluation conditions. Benchmark indicates which benchmark set was used, Variants indicates the types of variants included in the evaluation, and Evaluator indicates which tool did the evaluation.

**Condition**	**Benchmark**	**Variants**	**Evaluator**	**Notes**
WHC	GIAB v4.2.1	DeepVariant only	`whatshap`	Baseline approach used throughout main manuscript and this document
ASM	Assembly v0.9	DeepVariant only	`whatshap`	Shows impact of assembly benchmark set alone
ASM-all	Assembly v0.9	All variants	`whatshap`	Was not intended to evaluate larger variants
vcfdist	Assembly v0.9	DeepVariant only	`vcfdist`	Baseline vcfdist, useful for comparing impact of SVs and STRs in vcfdist-all
vcfdist-all	Assembly v0.9	All variants	`vcfdist`	Likely recommended approach in the future

Program 5: Command for running `bcftools` to merge variants from multiple sources into a single VCF prior to error evaluation. Variants are merged allowing for overlaps, then variants that are not evaluated are removed via `grep` to prevent downstream crashes. The remaining variants are normalized and sorted before recompression and indexing.

```
bcftools concat \
    --allow-overlaps \
    {input.vcf} \
    {input.pbsv_vcf} \
    {input.trgt_vcf} | \
grep -v "chrX\|chrY" | \
grep -v "SVTYPE=BND\\|SVTYPE=INV\\|SVTYPE=DUP" | \
bcftools norm -D \
    --fasta-ref {params.reference} | \
bcftools sort | \
bgzip > {output.vcf}
tabix {output.vcf}
```

Program 6: Command for running `vcfdist` to evaluate phasing results. The "-l 1000" limits the size of evaluated calls to prevent excessive runtimes.

```
vcfdist \
    {input.vcf} \
    {input.truth} \
    {params.reference} \
    -b {input.truth_bed} \
    -p {output.outdir}/ \
    -l 1000 \
    --max-threads {threads}
```

Table 8: Error metrics for alternative evaluation schemes on the HG002 Revio datasets. This table shows switches and flips for a few different evaluation techniques that are unpublished and in active development. The baseline, established method used throughout this document is `whatshap compare` (WHC). "ASM" uses an alternative assembly-based truth set that contains larger structural variants, but the evaluation is only performed on small variants. "ASM-all" uses the same alternative truth set, but also merges the in structural and tandem repeat variants before evaluation (HiPhase only). "vcfdist" uses the same alternative truth set and also the new `vcfdist` tool, which is designed to more accurately evaluate structural variant phasing. "vcfdist-all" additionally merges in structural and tandem repeat variants prior to evaluation (HiPhase only). **Bolded** values represent the best performance in the row.

Metric	WhatsHap	HiPhase (no SV)	HiPhase
Switches (WHC)	3,039	2,742	**2,523**
Flips (WHC)	1,805	274	**263**
Switches (ASM)	3,849	2,657	**2,530**
Flips (ASM)	11,081	**851**	1,047
Switches (ASM-all)	N/A	N/A	**2,527**
Flips (ASM-all)	N/A	N/A	**1,073**
Switches (vcfdist)	1,955	1,771	**1,521**
Flips (vcfdist)	2,023	1,005	**980**
Switches (vcfdist-all)	N/A	N/A	**1,528**
Flips (vcfdist-all)	N/A	N/A	**1,022**

Program 7: Command for running `samtools` to downsample an unaligned BAM file to an approximate, desired coverage level.

```
samtools view \
    --subsample {params.ds_rate} \
    --subsample-seed 1337 \
    --output {output.bam} \
    --output-fmt BAM \
    {input.bam}
```

3. Phasing with SVs and STRs reduces overall errors - When we compare HiPhase (no SV) to HiPhase on vcfdist, we see that switches decrease from 1,771 to 1,521 and flips decrease from 1,005 to 980. However, these errors only reflect the changes to the small variant VCF. If we instead compare to HiPhase on vcfdist-all, we see that 7 additional switches and 42 additional flips are found when SVs and STRs are included in the evaluation. Overall, this is a net reduction in switches with a slight gain in flips. This provides greater confidence that including SVs and STRs in the phasing improves the overall phase result while also provided downstream interpretation benefits.

2.5 Coverage experiment

We downsampled each of the HG002 datasets that were sequenced on the Revio system to approximately 5x, 10x, 15x, 20x, and 25x coverage levels. This process was performed by taking the mean coverage output from `mosdepth` and calculating the appropriate fraction to reach the desired depth. We then used the command in Program 7 to create an unaligned BAM of the approximate, desired coverage. We additionally merged each of the three replicates into three merged pairs (approximately 60x coverage each) as well as one merged dataset with all three datasets (approximately 90x coverage). Each downsampled and merged dataset was analyzed using the same process as the full samples including alignment, variant calling, phasing, and statistics gathering.

2.5.1 HiPhase coverage performance

Figure 3 shows the main results from this experiment. For HiPhase, the total number of flip errors was relatively stable, potentially showing a slight increase with coverage. Additionally, the total number of switch errors decreased rapidly from 10x to 20x, and then had a more gradual decrease as coverage increased. We observed a continuous increase in NG50 as coverage increased, with the most rapid gains at lower coverage levels. At lower coverage levels (¡15x), the gains are partly caused by an increasing accuracy of heterozygous variant calls from the upstream tools. However, beyond 15x, the gains are more likely from randomly acquiring longer reads that can span more distant gaps between heterozygous variants. This is best demonstrated by the merged datasets where NG50 continues to increase while showing no major gain in switchflip errors.

2.5.2 WhatsHap coverage performance

We also ran the same analysis using WhatsHap (also Figure 3). As coverage increased, we observed an increasing NG50 for WhatsHap as well, but at a rate slower than that of HiPhase. In constrast to HiPhase, the total number of flip errors increased rapidly from 5x to 30x before stabilizing. Additionally, the total number of switch errors was relatively stable across all coverages, but at a rate higher than HiPhase. We hypothesize that both the increasing flips and relatively slower increasing NG50 are caused by the downsampling component of WhatsHap. In summary, when compared to WhatsHap, HiPhase shows a large reduction in flip errors across all coverage levels, fewer switch errors especially at higher coverage, and an NG50 that is growing faster than WhatsHap as coverage increases.

2.6 Supplemental mapping experiment

Figure 4 shows the impact of using supplemental mappings to join phase blocks together into longer blocks. Using supplemental mappings is on by default in HiPhase, but that process can be disabled via the `--no-supplemental-joins` option. In general, there is a small increase in switchflip errors (< 10 in all samples) accompanied by an increase in NG50 when supplemental mappings are used. Overall, there was a $< 1\%$ increase in switchflips and $\approx 4\%$ increase in NG50 when using supplemental mappings.

2.7 Phased variant differences

While the summary of phased variants from Table 5 is useful for quick comparison on structural and tandem repeat variants that HiPhase is phasing, it hides details about which types of small variants are getting phased differently between HiPhase and WhatsHap. This is further complicated by the two tools having separate mechanisms for counting the variants that are phased, as well as conditions where a variant call is intentionally left unphased (i.e., ignored). We wrote a simple Python script that iterates over the phased small variant VCF files from WhatsHap and HiPhase in parallel, classifying each variant based on the type (SNV, insertion, deletion, or multi-allelic) and which tools had phased the variant.

Table 9 contains results for this experiment for the three Revio HG002 replicates. We note that HiPhase intentionally ignores variant calls that are fully contained within a tandem repeat call, as these are typically less accurate than a complete tandem repeat call. There are approximately 156K variants that are not assessed by HiPhase due to tandem repeat overlap, indicated by flagging them with "TR_OVERLAP" in the VCF. WhatsHap intentionally ignores multi-allelic variant calls, whereas HiPhase phases 232K of these variants. HiPhase also phased 70K structural variants and 204K tandem repeat variants, which were not assessed with WhatsHap. Across the remaining variants that are assessed by both tools, the vast majority are phased by both. HiPhase phased 41K more SNVs but about 19K fewer insertions and 21K fewer deletions than WhatsHap.

3 Result metrics

All metrics were gathered using a benchmarking pipeline built with `snakemake` (Mölder et al., 2021). Where possible, metric gathering commands were homogenized. The following sections describe the overall summary of the metrics followed by specific commands as `snakemake` templates and highlight tool-based differences where appropriate.

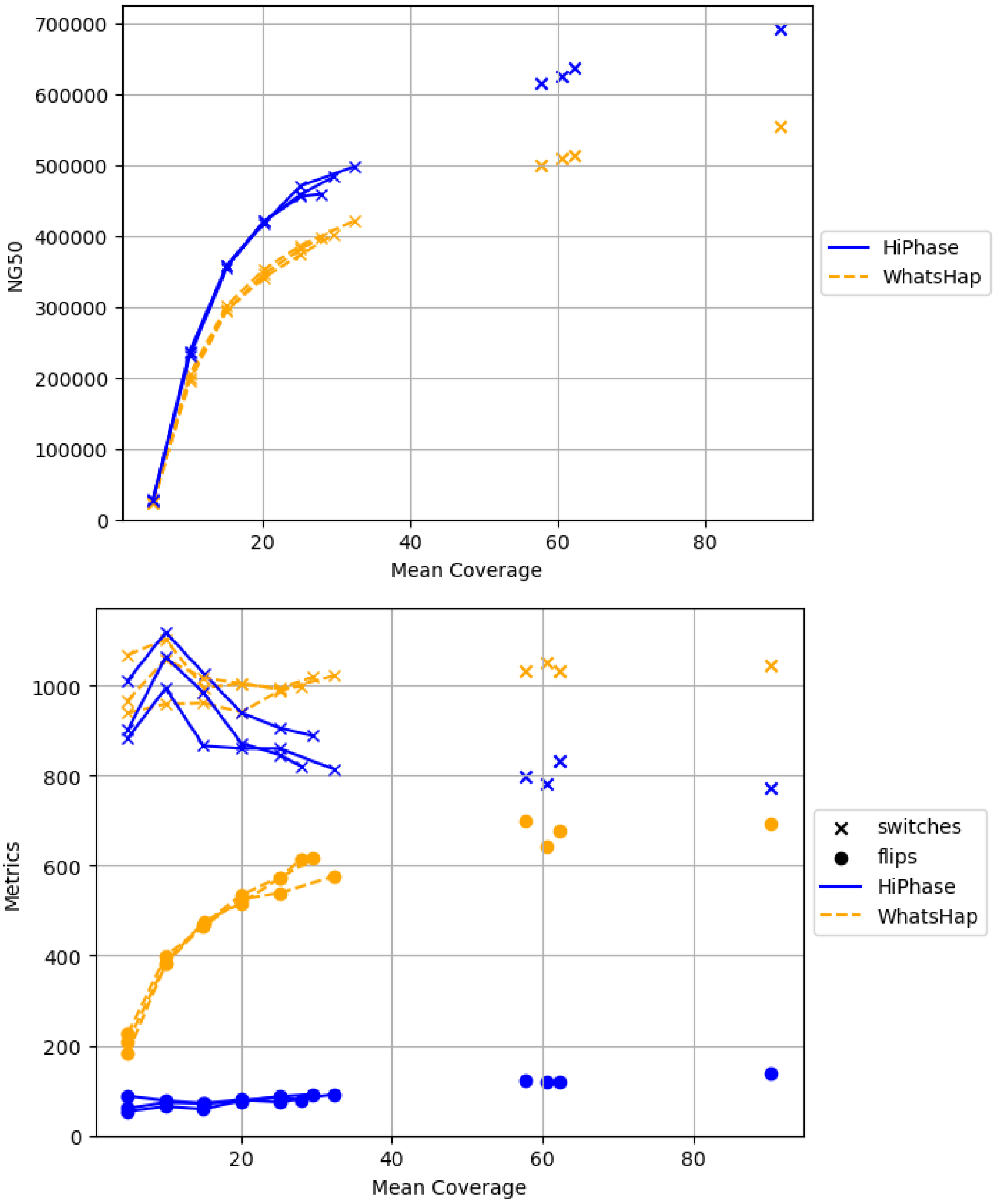

Figure 3: Coverage experiment results. The above figures show the NG50, switches, and flips for each of the downsampled and merged datasets. Points connected with a line represent a single replicate of HG002, while standalone points indicate a merged dataset (three datasets merging two replicates and one dataset merging all three replicates). For HiPhase, as coverage increases, we observe a steadily increasing NG50, relatively stable flip count, and decreasing switch count. For WhatsHap, NG50 is also increasing but at a slower rate than HiPhase, the total number of flip errors seems to increase rapidly up to 30x before stabilizing, and switch errors seem relatively stable at a rate higher than HiPhase.

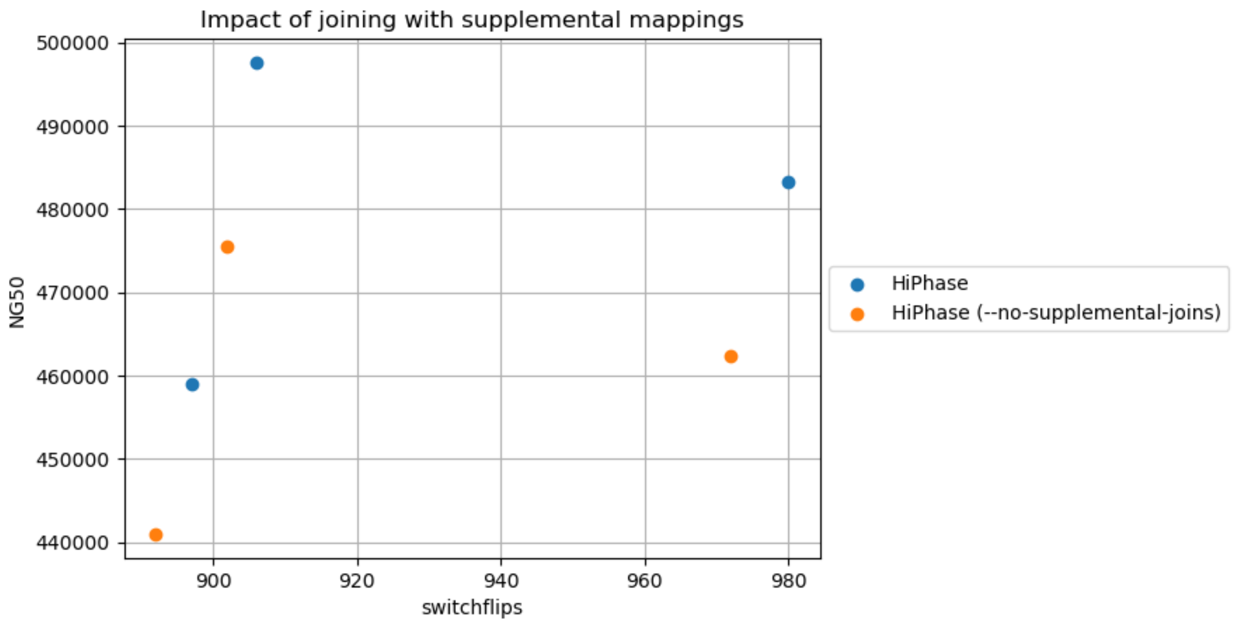

Figure 4: HiPhase supplemental mapping experiment results. The above figures show the NG50 and switchflips for each of the HG002 replicates under the default HiPhase conditions and with `--no-supplemental-joins`, disabling the using the supplemental mappings to create larger blocks.

Table 9: This table shows the final phased status for the small variant files from the three Revio HG002 replicates when run with WhatsHap and HiPhase. Note that HiPhase will intentionally ignore small variants that are fully contained within tandem repeats as these are typically less accurate than a complete tandem repeat call. Additionally, WhatsHap will intentionally ignore multi-allelic variation (leaving it unphased), so these are represented as "–" in the table. HiPhase is the only tool where SVs and STRs were assessed. Excluding multi-allelic sites, we note that the vast majority of each variant type is phased with both tools.

**Phasing status**	**SNV**	**Insertion**	**Deletion**	**Multi-allelic**	**SV $>$ 50bp**	**STR**
Both unphased	349,159	5,908	7,352	104,029	7,852	7,410
WhatsHap phased, HiPhase ignored	14,795	76,944	64,450	–	–	–
WhatsHap phased	103,776	23,645	25,811	–	–	–
HiPhase phased	144,313	4,045	4,849	232,685	33,482	204,783
Both phased	7,142,767	657,810	697,907	–	–	–

3.1 Metric definitions

3.1.1 Error metrics

These metrics are generated when comparing a phase result to the GIAB benchmark sets:

- Switchflips - A "switch" is when a pair of consecutive heterozygous variants in the phase result are incorrectly phased relative to the benchmark set, and a "flip" is two back-to-back switches such that only one variant is on the incorrect phase block. "Switchflips" is the total number of switches and flips across all phase blocks (switches are not double-counted in a flip). Typically, this metrics is thought of as the *number* of errors contained in the phase result.

- Hamming distance - The minimum number of phase orientations that need to be "flipped" in the phase result to exactly match the benchmark set. This metric is heavily influenced by block length, number of phased variants, and the location errors. We think of this as a measure of the *severity* of errors in the phase result. In internal testing, we found this metric to be highly variable for an individual dataset. Minor changes in switchflips could lead to much larger changes (both increases and decreases) in the measured hamming distance. In aggregate, it is often useful for assessing trends in error severity. On an otherwise correct phase block with V variants, one flip always increases hamming distance by 1 whereas one switch may increase the hamming distance by up to $\frac{V}{2}$.

Additional details on each can be found here: https://whatshap.readthedocs.io/en/latest/guide.html#whatshap-compare

3.1.2 Phase block metrics

These metrics are used to aggregate phase block statistics. For an individual phase block, the length is measured in base pairs (bp) from the position (POS field in VCF) of the first phased variant to the last phased variant (inclusive):

- NG50 - The smallest phase block length such that all blocks of this size or larger span at least 50% of the full genome (GRCh38) length.

- NGC50 - "Corrected NG50", this metric combines *length* with *accuracy*. Phase blocks are first split wherever a switchflip error occurs and then NG50 is re-calculated on the collection of smaller, error-free sub-blocks. Anecdotally, this metric is useful for defining trade-offs between reducing errors and increasing phase block length (as in "WhatsHap (optimized)" mode).

- Number of phased variants - The number of phased variants across all phase blocks, usually separated by variant type.

- Fully phased genes - The percentage of RefSeq genes where all heterozygous small variants are fully spanned by a single phase block.

3.2 Error metric command templates

Error metrics were gathered in a *homogenized* way by running `whatshap compare` against the corresponding benchmark set. Program 8 is the command template used in our benchmarking pipeline.

Key outputs from this command are:

1. Metrics `switches`, `flips`, and `switchflips` - the column `all_switchflips` from {`output.tsv`} is parsed into separate switch and flip categories and the result for all chromosomes are added together into a single value for the dataset; i.e. `switchflips = switches + flips`

2. Metric `hamming_distance` - the column `blockwise_hamming` from {`output.tsv`} is parsed and the result for all chromosomes are added together into a single value for the dataset

3. The location of each switch error is in {`output.error_bed`}.

Program 8: Command for running `whatshap compare` to gather error metrics on both WhatsHap and HiPhase outputs.

```
whatshap compare \
  --tsv-pairwise {output.tsv} \
  --switch-error-bed {output.error_bed} \
  {input.truth} \
  {input.vcf}
```

Program 9: Command for running `whatshap compare` to gather phase statistics on WhatsHap phase results.

```
whatshap stats \
  --tsv {output.tsv} \
  --block-list {output.blocks} \
  {input.vcf}
```

3.3 Phase block metric command templates

Metric gathering tools from WhatsHap are limited in that they cannot capture phase results from structural variants or multi-allelic variation. For WhatsHap results, this is not a problem because it does not attempt to solve those variant types. However, HiPhase is capable of including those types in the phase result, so we had to build the results into the HiPhase outputs in order to accurately capture them. The following sections describe how each phase block metric was gathered for each tool.

3.3.1 NG50 and number of phased variants

WhatsHap For WhatsHap, we used the built-in `stats` command to gather summary results. Program 9 contains the command template for this tool.

Key outputs from this command are:

1. Metrics `NG50` and number of phased variants - for both metrics, we parsed row `ALL` from {`output.tsv`} and collected columns `block_ng50` and `phased`, respectively.
2. The location of each phase block is in {`output.blocks`}.

HiPhase For HiPhase, we used the built-in options to gather summary results while phasing (Program 10).

Key outputs from this command are:

1. Metrics `NG50` and number of phased variants - for both metrics, we parsed row `all` from {`output.summary`} and collected columns `block_ng50` and `num_phased`, respectively.
2. The location of each phase block is in {`output.blocks`}.

Initially, we were using the same command from Program 9 to gather statistics for HiPhase results. However, WhatsHap does not correctly assess multi-allelic sites, a problem shared in both the `phase` and

Program 10: Partial command for running `hiphase` and collecting metrics at the same time.

```
hiphase \
  --blocks-file {output.blocks} \
  --summary-file {output.summary} \
  ...
```

Program 11: Command template for creating correctly phased sub-blocks from a tool's original full phase blocks.

```
bedtools subtract \
  -a {input.phase_block_bed} \
  -b {input.error_bed} > \
  {output.corrected_phase_block_bed}
```

the `stats` components of the tool. As a result, the `stats` command was consistently *under*-reporting the performance of HiPhase both in the number of variants and the phase block lengths, which led us to develop the custom statistic outputs that are built into HiPhase now. In general, the statistics from Programs 9 and 10 are strongly correlated but more correct with the HiPhase outputs.

3.3.2 NGC50 and "Fully phased genes"

Given the above output phase blocks (which are calculated differently), we *homogenized* the calculation of the NGC50 and the percentage of genes that were fully phased.

1. NGC50
   (a) Per dataset and method, we converted the reported phase block file (TSV/CSV) to a corresponding BED file by extracting the appropriate columns. This is {`input.phase_block_bed`} in Program 11.
   (b) The regions overlapping switchflip errors in the {`output.error_bed`} file, generated during Section 3.2, are removed from the phase block bed file using Program 11. This will split any phase blocks into sub-blocks that contain no errors relatively to the phase benchmark set.
   (c) We then re-calculated NG50 using the remaining, phased sub-blocks from {`output.correct_phase_block_bed`} to get NGC50.

2. "Fully phased genes"
   (a) We downloaded the latest RefSeq GRCh38 GFF3 file using this interface: https://www.ncbi.nlm.nih.gov/projects/genome/guide/human/index.shtml. Our downloaded version was annotated with "NCBI Homo sapiens Annotation Release 110".
   (b) We performed a one-time conversion of this file into a BED file, keeping only "gene" and "pseudogene" annotations from these sources: "BestRefSeq", "RefSeq", "Gnomon", "Curated Genomic", and "BestRefSeq,Gnomon". Additionally, we only kept annotations on primary chromosomes (all 22 autosomes, chrX, chrY, and chrM). This file is {`input.refseq_gene_bed`} in Program 12.
   (c) Per dataset and method, we converted the reported phase block file (TSV/CSV) to a corresponding BED file by extracting the appropriate columns. We then extended each phase block (both downstream and upstream) until it encountered another heterozygous variant in the dataset's small variant VCF file. These "extended" phase blocks represent the maximum extension of a phase block, intended to capture homozygous regions within genes. This is {`input.phase_block_bed`} in Program 12.
   (d) Per dataset, we ran `bedtools intersect` to capture all RefSeq gene regions that were fully covered by a single extended phase block using Program 12. The percentage fully phased was calculated by counting the number of remaining regions in {`output.intersection`} and dividing by the original count in {`input.refseq_gene_bed`} (e.g. `wc -l`). Steps (c) and (d) are captured in a single Python3 script that wraps `bedtools` and performs the calculations.

Program 12: Command template removing genes from a RefSeq BED file that were not fully covered by a single phase block.

```
bedtools intersect \
  -a {input.refseq_gene_bed} \
  -b {input.phase_block_bed} \
  -f 1.0 -wa -A > \
  {output.intersection}
```

3.3.3 Number of phased structural and tandem repeat variants

HiPhase is the only approach that phases structural and tandem repeat variants (SVs and STRs) in our tested method. To gather the number of phased SVs and STRs, we initially ran `whatshap stats` as in Program 9, but this produced incorrect results. Instead, we developed a custom Python3 script that parsed the VCF using `cyvcf2` (Pedersen and Quinlan, 2017b), counted variants with the appropriate statuses, and produced a JSON file. An example of the output is shown in Program 13. Note that `pbsv` produces structural variant calls down to approximately 20 bp, so there is some overlap between variants from DeepVariant and `pbsv`. In addition to unfiltered metrics, the output will provide the number of heterozygous sites and phased sites with length greater than the 50 bp size threshold (`_st` "size threshold" postfix), which is what was counted in our primary analyses.

Program 13: Example JSON output for structural variant statistics gathering.

```
{
    "heterozygous": 32223,
    "heterozygous_st": 14319,
    "phased": 25459,
    "phased_st": 11270,
    "singletons": 988,
    "size_threshold": 50,
    "unphased": 6764,
    "variants": 51814
}
```

3.4 Computational resource metrics

We have documented computational resource usage for the tested tools in Table 10. All results were gathered using the built-in `benchmark` option of snakemake (Mölder et al., 2021), details on this functionality can be found at this link: https://snakemake.readthedocs.io/en/stable/tutorial/additional_features.html#benchmarking. For our purposes, we gathered the max memory usage (`max_rss`), wall clock time (`s`), and CPU time (`cpu_time`) for a process. For HiPhase, a single job was used to run each dataset and 16 threads were allocated per job, so those results are simply copied directly from the benchmark outputs and merged into a single value for the method. Unfortunately, WhatsHap does not have built-in parallelization. For efficiency, we split the phasing into separate cluster jobs by chromosome (25 total sub-jobs: 22 for the autosomes plus chrX, chrY, and chrM) and then merged the results. This required us to also merge the compute resources used from benchmarking as follows:

- Wall clock time - *max* of all WhatsHap sub-jobs' wall clock time; if innately parallelized by chromosome, the longest single-chromosome run-time would be the limiting factor
- CPU time - *sum* of all WhatsHap sub-jobs' CPU time; if innately parallelized, the program would still theoretically use the same amount of CPU (and potentially more from overhead)
- Max memory usage - *max* of all WhatsHap sub-jobs' max memory usage; if innately parallelized, this would likely be higher due to parallel processing and memory consumption (e.g., summation); however,

Table 10: Compute resources required for each method. Best results in each column are **bolded**. Note that these resources are cumulative (e.g., summation) for all datasets in the corresponding category. Additionally, compute and memory resources were merged separately for WhatsHap due to the lack of innate parallelism. We note that these differences make it difficult to fairly compare the wall clock time and memory usage of these two tools. In general, "HiPhase (no SV)" used less compute but more memory than either WhatsHap approach. The "HiPhase" condition increased the compute costs above that of WhatsHap, and further increased max memory consumption as well.

System	Method	Wall clock time (sum, seconds)	CPU time (sum, seconds)	Max memory usage (sum, GB)
Sequel II	WhatsHap	**8,092**	87,961	**5.2**
	WhatsHap (optimized)	9,340	102,694	7.0
	HiPhase (no SV)	8,813	**45,311**	12.6
	HiPhase	13,956	102,576	18.7
Revio	WhatsHap	**8,504**	91,093	**5.7**
	WhatsHap (optimized)	9,440	113,074	8.0
	HiPhase (no SV)	9,016	**40,575**	13.7
	HiPhase	11,646	98,916	26.2

it is difficult to fairly assess this, so we left it as the maximum which is likely a lower bound for a parallelized WhatsHap

We did not penalize WhatsHap for the overhead of splitting and merging the input and output files, but there was additional compute associated with those steps. Additionally, we note that we ran these analyses in a cluster setting. Readers should be aware that all metrics, especially wall clock time, may reflect cluster congestion or other factors that are difficult to isolate in a cluster environment.

4 Deep Methods

NOTE: This section is intended to provide deeper information on how HiPhase methods work. Some sections from the main document are repeated here for continuity for the reader.

At a high level, the phasing problem can be broken apart into three major components: phase block generation, allele assignment, and diplotype solving. Phase block generation is the process of generating putative phase blocks by looking for pairs of adjacent heterozygous variant calls that are overlapped by at least one read mapping. These can be chained together to form a candidate phase block, and each one can be processed independently with respect to the next two components. Allele assignment is the process of converting all read mappings (i.e., observations) within a putative phase block into allelic observations, which are chains of reference or alternate alleles corresponding to the observed variants within the particular read mapping. Diplotype solving is the process of distilling these allelic observations into two representative haplotypes that are *expected* to complement each other (i.e., where one is the reference allele, the other is the alternate allele). In the following sections, we describe each of these components in greater detail, focusing on similarities and differences with existing approaches.

4.1 Phase block generation

Phase block generation is the process of generating putative phase blocks by looking for pairs of adjacent heterozygous variant calls that are overlapped by at least one read mapping. At a high level, a phase block starts by taking the first (or next) available variant on a chromosome and creating a single-variant block (or "singleton" block) from it. Then, the next variant is loaded and HiPhase searches for mappings that span both that new variant and the existing block. If no mappings span both, the algorithm will then check for supplementary mappings from the new variant into the existing block. To our knowledge, this supplementary mapping check is unique to HiPhase, and allows it to span coverage gaps caused by things like homozygous deletions and reference gaps. If at least one spanning (or supplementary) mapping is identified, then the

Program 14: Generic outline of how allele assignment works. An observed sequence (1) is checked for each allele (2-4) and alleles are stored sequentially (5). These are condensed into a final integer representation where only the alleles are left (6).

```
1 Sequence  : ACGAGTTTA
2 Pos 3 A>G :   G  | |  ALT
3 Pos 6 T>C :      T |  REF
4 Pos 8 C>G :        T  AMBIGUOUS
5 Alleles   : --1--0-2-
6 Condensed : 102
```

variant is joined to the current phase block and the process is repeated with the next variant. If no spanning (or supplementary) mappings are found, the current block is returned as a putative phase block and a new single-variant block created with the new variant. We note that HiPhase has parameters to adjust the default behavior for phase block generation that is described above.

Each putative phase block acts as an isolated sub-problem in the full solution. Because each putative block is unconnected by the read mappings at their ends, they represent a lower-bound on the number of phase blocks in the final solution (note: HiPhase assumes no phase block overlaps). Most importantly, each sub-problem can be solved independently, allowing for the remaining steps (allele assignment and diplotype solving) to be performed in parallel for each putative phase block. This forms the basis for multi-threading in HiPhase.

4.2 Allele assignment

Allele assignment is the process of converting all read mappings (i.e., observations) within a putative phase block into condensed allelic observations, which are chains of reference or alternate alleles corresponding to the observed alleles within the particular read mapping. The main idea is to simplify a long-read sequence (e.g., >15 kb) down to a smaller set of integer values representing which alleles are present within the read. Typically, these assignments correspond to reference (REF) or alternate (ALT) alleles, but we also allow for ambiguity, unassigned values, and multi-allelic variation (two ALT alleles at one position). Additionally, each observed allele is assigned a "quality" or "weight" indicating the cost to alter or ignore that allele in the diplotype solving process. Program 14 shows a simple example of converting an observation to its condensed representation.

To our knowledge, HiPhase is unique in that it has two modes for allele assignment: local re-alignment and global re-alignment. In brief, local re-alignment assigns alleles and quality based on a small window around each variant position (conceptually similar to the allele assignment process of WhatsHap (Patterson et al., 2015)). In contrast, global re-alignment will *fully* re-align the mapping against a local alt-aware reference graph using a graph-aware version of the wavefront algorithm (Marco-Sola et al., 2020). In general, local re-alignment is a faster process, but it is ill-suited for accurate allele assignment for large structural variants and some indels. Global re-alignment tends to be slower but is more accurate when it comes to allele assignment, especially in structural variants. HiPhase implements a "dual mode" allele assignment where if global re-alignment is too slow, it will fall back on local re-alignment for the phase block.

Once all mappings have been converted to a condensed allele representation, HiPhase has one final step where mappings with the same read name are collapsed into a single entry. The primary purpose of this step is to create a bridge between supplementary mappings that span a gap in coverage. This allows HiPhase to cross deletion events and reference gaps with split read mappings covering them. If the mappings for one read overlap but have a conflicting allele assignment, then that allele is converted to an ambiguous allele assignment in the collapsed representation. In the end, each read is represented exactly once in the collection of condensed alleles for the phase block.

4.2.1 Local re-alignment

Local re-alignment is an approach that is very similar to the allele assignment algorithm used by WhatsHap (Patterson et al., 2015). For an individual variant, local re-alignment takes the sequence overlapping the variant with a surrounding window ($\pm W$ base pairs) and performs two alignments to two different sequences: one version that matches allele 0 (typically REF allele) and one version that matches allele 1 (typically ALT allele). Whichever alignment produces the lowest cost (e.g., smallest edit distance) is selected as the allele for that variant. In the event of a tie, they are equidistant, and the allele is marked as ambiguous.

In HiPhase, the default window size is $W = 15$. Additionally, the tool will truncate this window if it overlaps other known variants from the VCF or if it detects unknown variant interference (e.g., a large insertion in the sequence). During development, this tended to produce more accurate allele assignments and reduce ambiguity in the assignments as well.

Once an allele is assigned, it is also given a quality value corresponding to the confidence that the allele is correct. For small variants (SNVs and indels), this quality value is a function of the base quality of the bases in the window that is then scaled depending on the variant type. For example, SNV variants tend to be the most accurate variant calls so they are given increased weight. Insertions, deletions, and indels are all down-weighted with respect to SNVs because they tend to generate more false positive calls. For HiPhase, these static weights were set via heuristics and may benefit from further tuning or some other form of dynamic weighting in the future.

Local-realignment tends to be ill-suited for larger structural and tandem repeat variants. For different mappings, a large variant can have drastically different coordinates due to local sequence similarity and subtleties in mapping. For window-based approaches, this makes it much easier to miss a true non-reference allele and incorrectly label it as reference allele. While we do not recommend using local re-alignment for phasing structural or tandem repeat variants, it is currently supported by HiPhase (partially because of dual mode allele assignment). Structural variant insertions and tandem repeat variants are handled in an identical manner to small insertions using the local window with realignment. Structural variant deletions use an overlap scoring scheme to determine whether an event is present or not, similar to how CNV benchmarking tools like Truvari (English et al., 2022) determine whether a variant call matches. In short, it counts the number of deleted bases in the mapping that overlap the deletion call. If a sufficient number of bases are marked as deleted in the mapping, then it will assign it the deletion allele. The quality of this event then scales based on how well it overlaps (e.g., if there is a 70% match, it will receive 70% of the maximum quality). Given the inaccuracy of structural variant local re-alignment, these events are down-weighted relative to other variant types.

4.2.2 Global re-alignment

To our knowledge, the application of global re-alignment to phasing is novel and unique to HiPhase. In contrast to local re-alignment, this approach attempts to fully re-align the entire read mapping against an ALT-aware graph sequence that is localized to the mapping. Using the local reference genome as a backbone for the graph, all provided variants (including homozygous variants that are not part of the phasing problem) are added sequentially to the graph structure. This approach allows methods like Partial-Order Alignment (POA) (Lee et al., 2002) to run on top of the graph. The core idea is to find the lowest cost path through the graph while also tracking the nodes (which correspond to alleles) that were traversed in that path. For those unfamiliar with POA, the Simpson Lab provides an intuitive explanation for those who are already familiar with pairwise alignment algorithms: `https://simpsonlab.github.io/2015/05/01/understanding-poa/`.

Unfortunately, the original POA approach can be quite slow on reads that are >10 kb in length due to scaling off of both the graph size and mapping length ($O(G * N)$ where G is the graph size and N is the mapping length). Additionally, we know that the graph and the mapping *should* be very similar since the read mapped to that location, a property that is not taken advantage of in the original POA approach. In contrast, the pairwise WFA algorithm (Marco-Sola et al., 2020) is specifically designed to leverage sequence similarity between two sequences, with a run-time of $O(N * s)$ where s is the number of differences between the two sequences. However, this algorithm was not designed to run on a graph structure.

HiPhase combines the benefits of POA and WFA into a novel implementation of the WFA algorithm that is designed to run on a localized reference graph structure. This algorithm leverages the benefits of WFA (specifically, scaling off of the number of differences, for run time of $O(G * s)$), while simultaneously tracking

the optimal series of nodes that were traversed in the graph. The result is an efficient, lowest-cost traversal of the localized graph that identifies the optimal allele assignments for the read mapping. We refer to this method as "graph WFA" in subsequent sections.

In theory, one could perform a full backtrace of the graph WFA algorithm to get base-level quality values, but this can be expensive and tricky to handle when ambiguity in node traversal is present in the re-alignment (e.g., an indel assignment is truly ambiguous, but different lengths of read sequence match the two options). Instead, global re-alignment in HiPhase uses *only* the variant type to determine allele quality scores. In general, the relative weights used by global re-alignment match those from local re-alignment (e.g., SNV has the highest weight, while indels are down-weighted relative to SNVs). As noted for local re-alignment, these static weights were set via heuristics and may benefit from further tuning or some other form of dynamic weighting in the future.

While this algorithm is generally fast for most phase blocks, it can still run prohibitively long in noisy areas or regions with lower accuracy in variant calling (these both increased the error term, s, above). To prevent excessive run-times, HiPhase enforces a per-block, CPU-time limit for global re-alignment. In the event that the user-provided CPU-time is exceeded for a given phase block, any work so far will be discarded, and the algorithm will revert to local re-alignment for *all* mappings in the block. This ensures that all allele and quality assignments for a given block use the same approach. In our test cases, the vast majority (>99.8%, see Table 12) of phase blocks succeed in global re-alignment.

We note that while the HiPhase implementation of a graph-based WFA approach was done in parallel to and without knowledge of GWFA, the core algorithms converged on very similar solutions. While the application and implementation of the algorithm is slightly different, we recommend reviewing the source code (`https://github.com/lh3/gwfa`) and the virtual seminar by author Heng Li (`https://www.youtube.com/watch?v=um_q8-BOXpg`) for greater details on core concepts for graph WFA.

4.2.3 Collapsing mappings

Once all mappings have been converted to their corresponding condensed allele representation, HiPhase has one final step where mappings with the same read name are collapsed into a single entry. The primary purpose of this step is to create a bridge between supplementary mappings that span a gap in coverage. This allows HiPhase to cross many homozygous deletion events and reference gaps despite lacking a single mapping that directly spans the gap. If conflicting alleles are encountered during this process (i.e., the mappings overlap and have a different assigned allele), then the allele is converted to an ambiguous representation. Figure 5 shows an example where this process allows HiPhase to create a single phase block spanning a reference gap where WhatsHap created two separate blocks. At the end of the collapsing process, each read is represented exactly once in the collection of condensed alleles for the phase block.

4.3 Diplotype Solving

Diplotype solving is the process of distilling the condensed allelic observations into two representative haplotypes that are *expected* to complement each other (i.e., where one is the reference allele, the other is the alternate allele). For our purposes, we define the core diplotype solving problem as a slight reformulation of the weighted minimum error correction (wMEC) problem as described by the authors of WhatsHap (Patterson et al., 2015). Given a matrix where each row corresponds to the condensed allele representation of one read in the phase block, the goal is to find two haplotypes, h_1 and h_2, such that the cost of changing each row to exactly match *either* h_1 or h_2 is minimized. Additionally, each allele has a cost or "weight" associated with altering that allele. We refer the reader to (Patterson et al., 2015) for greater technical details around this and other formulations of the phasing / wMEC problem.

4.3.1 A* phasing algorithm

HiPhase uses a version of the A* search algorithm (Hart et al., 1968) to solve the phasing problem. In general, A* search algorithms explore some search space by iteratively expanding the current lowest cost option (similar to Dijkstra's algorithm (Dijkstra, 2022)). The key difference with A* is that cost for a partial solution or "node" n is defined by both an observed cost, $g(n)$, and a heuristic estimated cost to reach the final goal, $h(n)$. As long as the heuristic is "admissible", meaning it never *over*-estimates the cost to the goal,

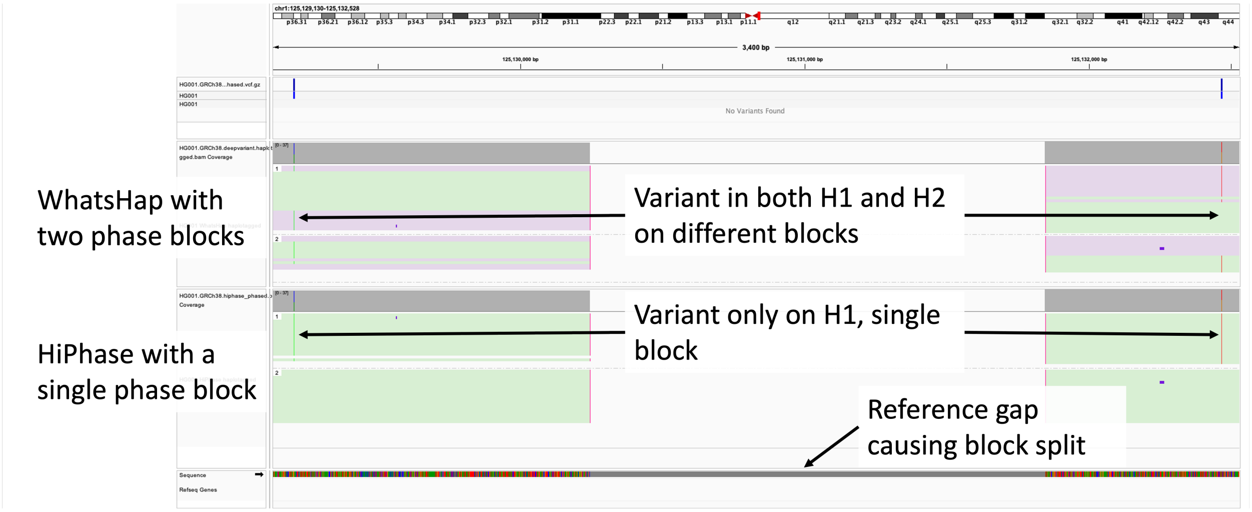

Figure 5: IGV screenshot of a reference gap in HG001. The top haplotagged BAM track is from WhatsHap, and the bottom haplotagged BAM track is from HiPhase. Mappings in both tracks are grouped by haplotag ID (HP:1 or HP:2) and colored by phase set (PS). In the WhatsHap track, there are two blocks corresponding to upstream (green) and downstream (purple) phase blocks. Read mappings have supplementary mappings across the gap but are assigned to different blocks and haplotypes. Additionally, heterozygous ALT variants immediately upstream and downstream do not cleanly segregate into only one haplotype, appearing in both HP:1 and HP:2 depending on the assigned block ID. In contrast, HiPhase has a single phase block (green) spanning the region, and both heterozygous ALT alleles are cleanly assigned to HP:1.

A* is guaranteed to find the optimal solution. Thus, the key challenges in all A* search algorithms are 1) defining the search space, 2) defining the cost structure, and 3) defining an efficient, admissible heuristic that is *ideally* close to the true cost. We break down each of these components for HiPhase in the following sections.

4.3.2 Defining the search space

Given our wMEC problem formulation, the search space is relatively simple: we are searching through all possible combinations of h_1 and h_2. Conceptually, this can be visualized as a search tree where the root contains empty haplotypes (i.e., $h_1 = h_2 =$ “”). All unexpanded nodes (initially just the root) are added to a priority queue that is based on the lowest cost (defined by $g(n) + h(n)$). In each iteration, the lowest cost node is removed from this queue and expanded. This expansion process adds one new node for each possible extension of the partial solution represented by the current lowest code node. In the expected case, this would be one of two phase orientations: $0|1$ and $1|0$. However, we also allow for variants to be converted to homozygous if that leads to a more optimal solution: 0/0 or 1/1 (in output files, these are left as unphased heterozygous calls, 0/1). This leads to a total of four expansions per node (two heterozygous and two homozygous). Given these expansion definitions and the initial empty root node, we can also say that each node at depth D contains only haplotype combinations of length D. Thus, for a phase block of length N, this tree has a *maximum* depth of N and has at most $O(4^N)$ nodes in a fully expanded tree. Finally, the first node at some depth, d, that is popped from the priority queue represents the optimal solution for the first d variants in the block. This is because all other nodes have a cost that is greater than or equal to this node, and due to the admissibility constraint of $h(n)$, we know that the costs of those nodes when extended can only go up. Thus, the first node in the traversal that is encountered at depth N is the solution to the full phase block.

In practice, we found that the majority of the phase blocks have very clean solutions, leading to near linear tree traversals (e.g., $O(N)$). However, some phase blocks have less clean solutions that create less direct, sometimes even exponential, tree traversals. These blocks tend to be clustered around problematic

genomic regions such as segmental duplications, low complexity regions, centromeres, or other high-mismatch regions. If left unchecked, they would likely have near-exponential run-times (e.g., $O(4^N)$) to reach the true optimal solution. To resolve this issue, we implemented a pruning strategy that limits the number of nodes in the priority queue. When the limit is reach, a pruning threshold, p, is incremented and all nodes that are not at least p deep in the tree are pruned. Functionally, this is pruning nodes that are relatively shallow in the tree exploration and less *likely* to contain the optimal solution. However, if *anything* is pruned this way, A* loses the guarantee of finding the optimal solution as the partial solution that was pruned may eventually lead to the true optimum. HiPhase tracks phase blocks where pruning occurs which may be useful for assessing phase block quality by users or downstream tools. On our test data, HiPhase has unpruned, guaranteed optimal solutions for approximately 88-93% of phase blocks depending on the dataset and allele assignment mode (see Table 12).

4.3.3 Defining the cost structure

When defining the cost of a given pair of partial haplotypes, we must compare those partial haplotypes to our condensed allele observations and the corresponding weights assigned to each allele. For a node n at depth d, we have two partial haplotypes of length d, h_{n1} and h_{n2}, that form partial candidate solutions for phasing the full block. Each read observation in the phase block is compared to the two haplotypes and assigned a cost of converting that read to have no conflicts with the haplotype. Then, the minimum of these two values is selected as the cost for this read and the node, effectively assigning it to one of the two partial haplotypes. The sum of all costs represents the total observed cost, $g(n)$, for node n. Mathematically, given a collection of reads, R, where each read has both alleles and weights, this can be represented with two formulas. The first formula (Equation 1) defines the cost function for an individual comparison of a read r to a partial haplotype h, and the second formula (Equation 2) defines the combined cost across the read collection when compared to both partial haplotypes associated with node n:

$$\text{cost}(r,h) = \sum_{i=0}^{|h|} \begin{cases} 0, & \text{if } r.allele[i] = h[i], \\ r.weight[i], & \text{otherwise} \end{cases} \tag{1}$$

$$g(n) = \sum_{r}^{R} \min(\text{cost}(r, h_{n2}), \text{cost}(r, h_{n1})) \tag{2}$$

While not specified in the above formula, all weights for ambiguous or unassigned alleles are set to 0, so there is no cost associated with reads that have unassigned alleles because they do not fully span a phase block. This means that once a node's haplotypes have extended past the last set allele for a read, the cost of that read becomes fixed for all extensions of that node (e.g., there is no weight on the read past that point that may change the minimum cost). In practice, this allows HiPhase to separate the cost of $g(n)$ into costs associated with "frozen" and "liquid" reads. Frozen reads will *never* change cost value as the haplotypes are extended, whereas liquid reads may change by the lowest cost flipping from one haplotype to the other. Frozen read costs are stored in aggregate and not recomputed with each extension to reduce compute time.

4.3.4 Defining the heuristic

Given the above definitions, the A* algorithm could run by simply setting the heuristic component to zero, $h(n) = 0$, which functionally leads to Dijkstra's algorithm (Dijkstra, 2022). However, this would be a very poor heuristic leading to over-traversal of the tree. Ideally, there is a heuristic that is very close to the actual final cost of the solution. While not guaranteed, heuristics closer to the actual cost *tend* to reduce run-time for most A* algorithms.

In HiPhase, we calculate the heuristic by solving sub-problems from the full block. Conceptually, if a phase block has N variants to phase, the block can be broken into sub-problems of some length, $S \leq N$. Any solution to the full phase block *must* also span the sub-problems as well. Let P_0 represent the first sub-problem and P_{N-S} be the last sub-problem and assume *some* algorithm can find an optimal solution to these sub-problems. One way to estimate the *minimum* cost of the full solution is to find a long chain of non-overlapping sub-problems from P_0 to P_{N-S}. The optimal solutions from these sub-problems can then be added together to form a full estimate. While these optimal sub-problem solutions may not be part of the

Table 11: Table showing a simplified version of how the heuristic chain is built to create heuristic estimated costs for all nodes at depth d. Sub-problem solutions are initial generated, in this case subproblems of length 2 (e.g. two variants phased together), and stored as P. Then the heuristic chain is constructed in reverse order. The last 2 nodes simply copy the subproblem solutions. Then, starting with variant C, the chaining process looks at both the subproblem solution and the chain solution that is 2 away ($h(E)$ for variant C). This process continues in reverse order until the full heuristic is solved starting at variant A. In practice, this forms a monotonically decreasing array for the heuristic costs, $h(d)$.

**Variant index, v**	**A**	**B**	**C**	**D**	**E**
**Subproblem cost, P_v**	3	2	5	4	0
**Heuristic cost, $h(d)$**	$h(A) = P_A + h(C) = 3 + 5 = 8$	$h(B) = P_B + h(D) = 2 + 4 = 6$	$h(C) = P_C + h(E) = 5 + 0 = 5$	4	0

final solution (they are locally optimal, not necessarily globally optimal), any solution to the full problem *cannot* create a solution with less cost than the chain of locally optimal subproblems (it may be equal). Additionally, this property is not limited to the full phase block but can be applied to any partial solution as well. For example, if we wanted to estimate the cost from P_q to P_{N-S}, we could run a similar chaining approach starting from P_q instead of P_0. This means that for any point in the full phase block, we have a general strategy to estimate the distance to the end of the block, which we use for our heuristic estimate, $h(n)$. In Table 11, we show a simplified example of the full heuristic being constructed for a toy problem.

There are several subtleties to how HiPhase does this in practice. First, given the above strategy, the heuristic estimates are tied to the variant index in the phase block, which is also the depth in the search tree (i.e., $h(n) = h(\text{depth of } n) = h(d)$). This means all nodes at a given depth, d, will have the same heuristic estimate to reach the end of the phase block. Second, HiPhase calculates the heuristic in reverse-linear order such that P_{N-S} is the first solved sub-problem and P_0 is the last. This allows HiPhase to build up the full heuristic chain as it goes (e.g., $h(q) = h(q + S) + P_q$). Third, while we focus on fixed-size sub-problems in our description, HiPhase will compute all sub-problems of size $\leq S$. This allows for chaining a different number of sub-problem solutions of potentially different sizes (e.g., 10 variants could be sub-problems of size $(5 + 5)$, $(3 + 3 + 4)$, etc.). Fourth, while the sub-problem solver is looking for *locally minimal* cost solutions, the heuristic is looking for the *maximum* cost chain of these minimal solutions. Intuitively, this is because if some chain of minimal sub-problem solutions exists with some cost, we know that any full phase block solution *must* have at least that cost and likely more from joining the full chain of sub-problem solutions together. Thus, it looks for the most costly chain of non-overlapping sub-problems to obtain the closest estimate to the actual cost. Finally, HiPhase uses a recursive A* phasing algorithm to solve the sub-problems. This recursive approach is generally identical to the full unpruned A* phasing algorithm, but it is limited to a fixed number of node expansions to reduce the computational burden of solving each sub-problem. This means that the heuristic will not always use the full sub-problem solution of length S, but may instead terminate early.

4.4 Algorithm Statistics

HiPhase can output statistics about each block and the performance of the underlying algorithms on that block. Table 12 shows some summary statistics on the allele assignment and A* algorithm execution for the datasets used in our results. Each statistic is briefly described here:

- Percent blocks globally re-aligned – When global re-alignment is enabled, this is the percentage of putative phase blocks that successfully finished the global re-alignment process within the allotted CPU time.
- {`variant_type`} REF:ALT ratio – For a given {`variant_type`}, this is the ratio of alleles that were assigned to the reference (REF) or alternate (ALT) allele. Ideally, this ratio is near 50:50, indicating an unbiased balance in allele assignment. Deviations from 50:50 may indicate errors in allele assignment and/or errors in upstream variant calling (e.g., false positives or inaccurate positions / ALT sequences).
- Heuristic / actual cost – Across all blocks, this is the total heuristic estimated cost divided by the actual cost in the final solutions. A perfect heuristic has a value of 1.0, indicating that it *exactly* estimated the

actual cost. While not a guarantee, as this value approaches 1.0, the A* algorithm *tends* to converge on a solution faster by exploring fewer nodes.

- Unpruned, exact solutions – The percentage of blocks that converged on a solution without performing any pruning of the search space. From an A* phasing perspective, these solutions are *guaranteed* to be optimal given the problem design. Note that optimal does *not* always mean biologically correct, but we expect these to be correlated given the problem design.

Table 12 includes a comparison of three modes of running HiPhase. "Small variants only" is the same as "HiPhase (no SV)" and "All variants (global, 300 sec)" is the same as "HiPhase" in the main manuscript. The "local" mode phases small, structural, and tandem repeat variants, but without global re-alignment enabled. As noted in Section 4.2.1, we do not recommend this mode for assigning structural or tandem repeat variant alleles but include it here for demonstrating the benefits of global re-alignment mode.

Table 12 highlights a few notable differences between local and global modes. First, with global re-alignment, the ratio of alleles assigned to the ALT haplotypes improves for structural variant deletions, structural variant insertions, and tandem repeats, with the greatest improvement in insertions and tandem repeats. This is most likely because the global re-alignment process is more capable of handling noise in the mapping location of large insertions. Second, the number of exact solutions generated while using global mode increased slightly ($\sim$1%), indicating that the algorithm can more easily phase the blocks as a result of more accurate allele assignments. Finally, while global re-alignment can timeout in difficult regions, the vast majority of the phase blocks succeeded (>99.8%) within the given time limit.

Table 12: Algorithmic performance of the HiPhase implementation of global re-alignment and A* phasing on Sequel II system and Revio system datasets. The first three statistics are gathered from the allele assignment process, and the last two are from the A* phasing algorithm. Structural variant (SV) deletion and insertion ratios are separated to show the separate impact on each type. The best metric for each row is **bolded**.

System	Metric	Small variants only (local)	All variants (local)	All variants (global, 300 sec)
Sequel II	Percent blocks globally re-aligned	N/A	N/A	**99.89%**
	SV Deletion REF:ALT ratio	N/A	58.34 : 41.66	**56.47 : 43.53**
	SV Insertion REF:ALT ratio	N/A	72.66 : 27.34	**62.11 : 37.89**
	STR REF:ALT ratio:	N/A	84.56 : 15.44	**51.29 : 48.71**
	Heuristic / actual cost	**0.8766**	0.8725	0.8298
	Unpruned, exact solutions	93.08%	92.28%	**93.09%**
Revio	Percent blocks globally re-aligned	N/A	N/A	**99.85%**
	SV Deletion REF:ALT ratio	N/A	59.03 : 40.97	**57.61 : 42.39**
	SV Insertion REF:ALT ratio	N/A	74.68 : 25.32	**68.32 : 31.68**
	STR REF:ALT ratio	N/A	86.45 : 13.55	**52.06 : 47.94**
	Heuristic / actual cost	**0.8841**	0.8815	0.8352
	Unpruned, exact solutions	89.32%	88.51%	**89.68%**

References

E. W. Dijkstra. *A Note on Two Problems in Connexion with Graphs*, pages 287–290. ACM, jul 12 2022. doi:10.1145/3544585.3544600. URL http://dx.doi.org/10.1145/3544585.3544600.

E. Dolzhenko, A. English, H. Dashnow, G. De Sena Brandine, T. Mokveld, W. J. Rowell, C. Karniski, Z. Kronenberg, M. C. Danzi, W. A. Cheung, et al. Resolving the unsolved: Comprehensive assessment of tandem repeats at scale. *bioRxiv*, pages 2023–05, 2023.

T. Dunn and S. Narayanasamy. vcfdist: Accurately benchmarking phased small variant calls in human genomes. *bioRxiv*, 2023. doi:10.1101/2023.03.10.532078. URL https://www.biorxiv.org/content/10.1101/2023.03.10.532078v2.

A. C. English, V. K. Menon, R. A. Gibbs, G. A. Metcalf, and F. J. Sedlazeck. Truvari: refined structural variant comparison preserves allelic diversity. *Genome Biology*, 23(1), dec 27 2022. ISSN 1474-760X. doi:10.1186/s13059-022-02840-6. URL http://dx.doi.org/10.1186/s13059-022-02840-6.

P. Hart, N. Nilsson, and B. Raphael. A Formal Basis for the Heuristic Determination of Minimum Cost Paths. *IEEE Transactions on Systems Science and Cybernetics*, 4(2):100–107, 1968. ISSN 0536-1567. doi:10.1109/tssc.1968.300136. URL http://dx.doi.org/10.1109/TSSC.1968.300136.

C. Lee, C. Grasso, and M. F. Sharlow. Multiple sequence alignment using partial order graphs. *Bioinformatics*, 18(3):452–464, mar 1 2002. ISSN 1367-4811. doi:10.1093/bioinformatics/18.3.452. URL http://dx.doi.org/10.1093/bioinformatics/18.3.452.

S. Marco-Sola, J. C. Moure, M. Moreto, and A. Espinosa. Fast gap-affine pairwise alignment using the wavefront algorithm. *Bioinformatics*, sep 11 2020. ISSN 1367-4803. doi:10.1093/bioinformatics/btaa777. URL http://dx.doi.org/10.1093/bioinformatics/btaa777.

F. Mölder, K. P. Jablonski, B. Letcher, M. B. Hall, C. H. Tomkins-Tinch, V. Sochat, J. Forster, S. Lee, S. O. Twardziok, A. Kanitz, A. Wilm, M. Holtgrewe, S. Rahmann, S. Nahnsen, and J. Köster. Sustainable data analysis with Snakemake. *F1000Research*, 10:33, jan 18 2021. ISSN 2046-1402. doi:10.12688/f1000research.29032.1. URL http://dx.doi.org/10.12688/f1000research.29032.1.

M. Patterson, T. Marschall, N. Pisanti, L. Iersel, L. Stougie, G. W. Klau, and A. Schönhuth. Whatshap: Weighted Haplotype Assembly for Future-Generation Sequencing Reads. *Journal of Computational Biology*, 22(6):498–509, 6 2015. ISSN 1066-5277. doi:10.1089/cmb.2014.0157. URL http://dx.doi.org/10.1089/cmb.2014.0157.

B. S. Pedersen and A. R. Quinlan. Mosdepth: quick coverage calculation for genomes and exomes. *Bioinformatics*, 34(5):867–868, oct 31 2017a. ISSN 1367-4803. doi:10.1093/bioinformatics/btx699. URL http://dx.doi.org/10.1093/bioinformatics/btx699.

B. S. Pedersen and A. R. Quinlan. cyvcf2: fast, flexible variant analysis with Python. *Bioinformatics*, 33(12): 1867–1869, feb 6 2017b. ISSN 1367-4803. doi:10.1093/bioinformatics/btx057. URL http://dx.doi.org/10.1093/bioinformatics/btx057.

R. Poplin, P.-C. Chang, D. Alexander, S. Schwartz, T. Colthurst, A. Ku, D. Newburger, J. Dijamco, N. Nguyen, P. T. Afshar, S. S. Gross, L. Dorfman, C. Y. McLean, and M. A. DePristo. A universal SNP and small-indel variant caller using deep neural networks. *Nature Biotechnology*, 36(10):983–987, sep 24 2018. ISSN 1087-0156. doi:10.1038/nbt.4235. URL http://dx.doi.org/10.1038/nbt.4235.

J. Wagner, N. D. Olson, L. Harris, Z. Khan, J. Farek, M. Mahmoud, A. Stankovic, V. Kovacevic, B. Yoo, N. Miller, J. A. Rosenfeld, B. Ni, S. Zarate, M. Kirsche, S. Aganezov, M. C. Schatz, G. Narzisi, M. Byrska-Bishop, W. Clarke, U. S. Evani, C. Markello, K. Shafin, X. Zhou, A. Sidow, V. Bansal, P. Ebert, T. Marschall, P. Lansdorp, V. Hanlon, C.-A. Mattsson, A. M. Barrio, I. T. Fiddes, C. Xiao, A. Fungtammasan, C.-S. Chin, A. M. Wenger, W. J. Rowell, F. J. Sedlazeck, A. Carroll, M. Salit, and J. M. Zook. Benchmarking challenging small variants with linked and long reads. *Cell Genomics*, 2(5):100128, 5 2022. ISSN 2666-979X. doi:10.1016/j.xgen.2022.100128. URL http://dx.doi.org/10.1016/j.xgen.2022.100128.

J. M. Zook, D. Catoe, J. McDaniel, L. Vang, N. Spies, A. Sidow, Z. Weng, Y. Liu, C. E. Mason, N. Alexander, E. Henaff, A. B. McIntyre, D. Chandramohan, F. Chen, E. Jaeger, A. Moshrefi, K. Pham, W. Stedman, T. Liang, M. Saghbini, Z. Dzakula, A. Hastie, H. Cao, G. Deikus, E. Schadt, R. Sebra, A. Bashir, R. M. Truty, C. C. Chang, N. Gulbahce, K. Zhao, S. Ghosh, F. Hyland, Y. Fu, M. Chaisson, C. Xiao, J. Trow, S. T. Sherry, A. W. Zaranek, M. Ball, J. Bobe, P. Estep, G. M. Church, P. Marks, S. Kyriazopoulou-Panagiotopoulou, G. X. Zheng, M. Schnall-Levin, H. S. Ordonez, P. A. Mudivarti, K. Giorda, Y. Sheng, K. B. Rypdal, and M. Salit. Extensive sequencing of seven human genomes to characterize benchmark reference materials. *Scientific Data*, 3(1), jun 7 2016. ISSN 2052-4463. doi:10.1038/sdata.2016.25. URL http://dx.doi.org/10.1038/sdata.2016.25.
